# CRISPR-Cas9 in the Tailoring of Genetically Engineered Animals

**DOI:** 10.3390/cimb47050330

**Published:** 2025-05-04

**Authors:** Wiktoria Urban, Marta Kropacz, Maksymilian Łach, Anna Jankowska

**Affiliations:** Department of Cell Biology, Poznan University of Medical Sciences, 61-701 Poznań, Poland; wurban@ump.edu.pl (W.U.); marta.kropacz02@gmail.com (M.K.); maksymilianlach18539@gmail.com (M.Ł.)

**Keywords:** CRISPR-Cas9, genome editing, genetically engineered animals, model organisms, xenotransplantation

## Abstract

CRISPR-Cas9 enables targeted genome editing and has become a pivotal tool in biomedical research and animal genome engineering. This review highlights its application in generating genetically modified animals used as preclinical disease models, bioreactors for recombinant protein production, and potential sources of xenotransplantation organs. We also discuss its role in improving livestock traits, welfare, and breeding efficiency. The benefits and limitations of CRISPR-Cas9 are examined, emphasizing its transformative potential in research and agricultural biotechnology.

## 1. Introduction

The Clustered Regularly Interspaced Short Palindromic Repeats (CRISPR)-associated protein 9 (Cas9)—or CRISPR-Cas9—system, a transformative genome editing tool, has altered biological research. Initially identified in *Escherichia coli* by Ischino et al. in 1987 at Osaka University, CRISPR-Cas9 is a component of the bacterial adaptive immune system, enabling the sequence-specific targeting and cleavage of foreign DNA or RNA, thereby protecting bacteria from viral infection [1]. This system functions through the integration of viral DNA fragments, known as spacers, into the CRISPR array during the adaptation phase, effectively creating a genetic memory of past infections [2].

Leveraging this natural mechanism, researchers have adapted CRISPR-Cas9 for versatile genome modifications in eukaryotic cells, facilitating gene insertion, deletion, and sequence alteration [2,3,4,5].

To achieve functionality in eukaryotes, several key modifications were necessary, including the addition of nuclear localization signals (NLS), codon optimization, the design of synthetic single-guide RNAs (sgRNAs), and the development of effective delivery mechanisms [3,4,6,7,8,9,10,11]. A comparative overview of CRISPR-Cas9’s role as a bacterial immune system and its application as a genome editing tool is presented in Table 1.

**Figure 1:**
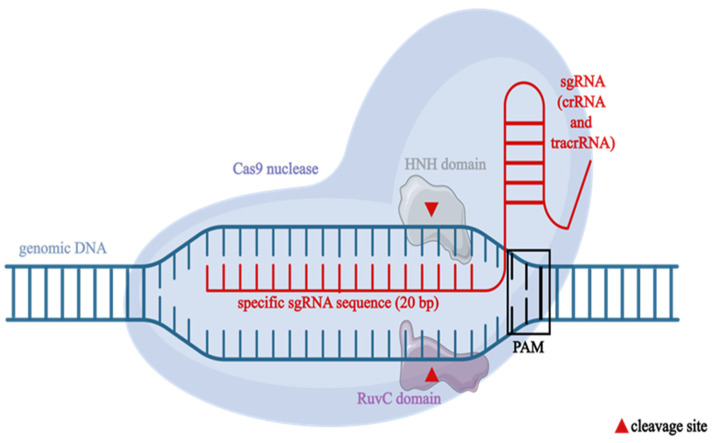
The CRISPR-Cas9 system, highlighting its key components and mode of action. The Cas9 nuclease is guided by a single-guide RNA (sgRNA), which combines CRISPR RNA (crRNA) and trans-activating CRISPR RNA (tracrRNA), to a complementary genomic DNA sequence adjacent to a protospacer adjacent motif (PAM). The Cas9 protein introduces a double-strand break via its two nuclease domains: the histidine–asparagine–histidine endonuclease domain (HNH), which cleaves the DNA strand complementary to the sgRNA, and the RuvC domain, which cleaves the non-complementary strand. This site-specific cleavage underpins the use of CRISPR-Cas9 in precise genome editing applications. Figure created with elements from BioIcons (CC 0) and Servier Medical Art (CC 3.0).

The mechanism of CRISPR-Cas9 acting as a gene editing tool relies on two crucial components (shown in Figure 1): (i) an artificially bioengineered single-guided RNA (sgRNA) with a sequence that matches a desired target gene and (ii) Cas9 (CRISPR-associated protein 9) endonuclease, which introduces double-stranded breaks (DSBs) in DNA [12]. Synthetic sgRNA, related to the bacterial crRNA/tracrRNA (CRISPR-RNA/tracrRNA—trans-activating CRISPR RNA) duplex, improves the target specificity while reducing off-target effects [8,9,10]. The Cas9 protein contains two nuclease domains: the crossover junction endodeoxyribonuclease RuvC (RuvC) and the histidine–asparagine–histidine endonuclease domain (HNH), recognizing DNA cleavage sites [13]. This site, typically 20 nucleotides long, is a target for RNA-guided endonuclease (RGEN). Bacterial Cas9 cannot reach the nuclei of eukaryotic cells. Thus, the protein was modified to contain NLS, mediating the transfer of proteins from the cytoplasm into the nucleus [7,10,14].

## 2. CRISPR-Cas9 Delivery to Animal Cells

CRISPR-Cas9 technology offers a flexible framework for the production of various genetically engineered animals; however, the efficacy of genome editing is primarily contingent upon the efficiency and accuracy of its delivery to target cells. Diverse strategies for CRISPR-Cas9 system delivery, dependent on the animal species, developmental phase, and intended modification, have been established. Each of these transfer modes possesses distinct advantages and constraints [15].

Direct delivery methods involve introducing the CRISPR-Cas9 components (as DNA, RNA, or protein) directly into zygotes or embryos. Microinjection, one of the most widely used techniques, enables precise delivery into the pronucleus or cytoplasm and has been successfully employed in generating genetically modified animals, such as mice, pigs, and non-human primates. While technically demanding, it allows for high editing efficiency and is suitable for introducing a wide range of modifications, including knockouts and precise gene insertions [15,16,17,18].

Electroporation offers a less invasive alternative by using an electric field to permeabilize the cell membrane, allowing CRISPR components to enter. This technique has gained traction due to its simplicity, reduced embryonic damage, and scalability, making it particularly useful in rodent models. However, it may be less effective for larger or more developmentally complex embryos [19,20,21,22].

Nanoparticle-based delivery systems provide a non-viral, scalable approach, minimizing potential immune responses and insertional mutagenesis. These are still under refinement for in vivo applications in larger animals, but they hold promise for future clinical and agricultural use [23,24].

Finally, viral vectors, such as lentiviruses and adenoviruses, offer high transduction efficiency and stable expression, especially in somatic tissue. They have been used to generate transgenic livestock, although concerns over immunogenicity and off-target integration persist [22,25,26].

The indirect delivery of CRISPR-Cas9, most notably the combination of microinjection with somatic cell nuclear transfer (SCNT), involves editing donor somatic cells ex vivo before transferring their nuclei into enucleated oocytes. This method allows for the clonal selection of edited cells before animal generation and is especially useful for species where direct embryo editing is inefficient or ethically constrained. SCNT-based CRISPR-Cas9 delivery has been applied to generate modified animals with traits relevant to research and agriculture [27,28,29].

Each method presents trade-offs between efficiency, technical complexity, cost, and applicability to different species. Table 2 provides a detailed overview of these delivery strategies.

## 3. CRISPR-Cas9 in Animal Genome Editing

**Figure 2:**
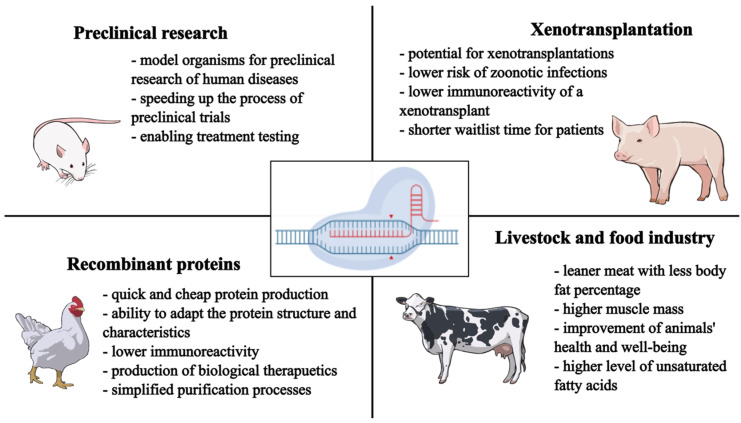
Key applications of genome editing technologies in animals across biomedical and agricultural fields. In preclinical research, genetically modified models facilitate the study of human diseases and accelerate therapeutic testing. In xenotransplantation, genome editing reduces the zoonotic risk and immunogenicity, improving organ compatibility. Recombinant protein production in animals enables the low-cost and efficient synthesis of therapeutic proteins with reduced immunoreactivity. In the livestock sector, genetic modifications enhance animal health, increase muscle mass, reduce fat content, and improve the nutritional value of animal products. These applications highlight the broad utility of CRISPR-Cas9-based genome editing in advancing biomedicine and agriculture. Figure created with BioIcons (CC 0) and NIAID BioArt (Public Domain).

The generation of transgenic animals employs various genome editing techniques, including CRISPR-Cas9, zinc finger nucleases (ZFNs), and transcription activator-like effector nucleases (TALENs). Among these, CRISPR-Cas9 has become the predominant method of producing transgenic animals. CRISPR-Cas9-mediated genome modifications facilitate the creation of transgenic animal models for diverse applications (Figure 2). These genetically modified animals serve as crucial tools for the development of genetic models of human diseases, enabling a deeper understanding of molecular pathogenesis and, subsequently, the advancement of novel therapeutic strategies [4].

The livestock industry leverages transgenic animals exhibiting desirable traits, including enhanced resistance to adverse environmental conditions and pathogens, accelerated growth rates, and improved efficiency in food production [60]. Transgenic animals may act as bioreactors, enabling the production of recombinant proteins, such as monoclonal antibodies, blood factors, albumin, enzymes, and vaccines. Finally, genetically modified animals are gaining interest as donors for xenotransplantation [18,61,62,63,64].

### 3.1. CRISPR-Cas9: Preclinical Animal Models

CRISPR-Cas9 has expanded the biomedical research capabilities by enabling precise and efficient genome editing in transgenic animal models. In conjunction with preclinical studies, these models have significantly advanced our understanding of human disease mechanisms and accelerated drug and vaccine development [65]. Cattle, sheep, pigs, mice, and rats are widely used in preclinical studies, but mice are preferred due to their rapid reproductive cycle and convenient maintenance [66]. Transgenic animal models are indispensable in oncology. Precise genome editing enables the creation of models exhibiting specific oncogenic traits, facilitating the investigation of gene knock-in or knock-down effects on cancer development, progression, and potential therapeutic reversal. CRISPR-Cas9-modified pigs, mice, and zebrafish serve as valuable preclinical models for diverse cancer types [67,68,69]. A notable example of such animal models is the K14-HPV16 transgenic mouse, designed to elucidate cervical carcinogenesis driven by human papillomavirus (HPV). In this model, CRISPR-Cas9-mediated mutations targeting the E7 gene effectively restored the expression of retinoblastoma protein (RB), E2F transcription factor 1 (E2F1), and cyclin-dependent kinase 2 (CDK2), thereby reversing the observed cervical carcinogenesis phenotypes [70]. Furthermore, large animal models are crucial in studying tumorigenesis and evaluating cancer therapies. Engineered pigs, known as oncopigs, provide a valuable model for hepatocellular carcinoma (HCC). Oncopig hepatocytes were used to create HCC in vitro via Cre recombinase. The CRISPR-Cas9 knockout of PTEN and CDKN2A increased cell migration and proliferation. Autologous liver injections produced localized tumors, while portal vein injections caused multifocal tumors that regressed. Genetic analysis confirmed PTEN and CDKN2A knockouts. This demonstrates CRISPR-Cas9’s ability to create tailored HCC tumors in swine via autologous implantation [69].

CRISPR-Cas9 models hereditary genetic diseases. Targeting the FAH gene for tyrosinemia type I (HTI) created HTI mouse models with renal and hepatic failure and obesity [71,72]. Gene editing reversed this, showing potential for HTI treatment [73].

CRISPR-Cas9 has also generated models for neurodegenerative diseases, such as Huntington’s disease, Duchenne muscular dystrophy, or Parkinson’s disease. Inactivating the mutated Huntingtin (HTT) gene reversed neuropathology and behavioral abnormalities, even in aged mice. This demonstrates that inhibiting mutant protein synthesis facilitates the clearance of accumulated toxic proteins and promotes neural repair [74]. In primates, CRISPR-Cas9 targeted the dystrophin gene, causing muscle tissue mutations in up to 87% of alleles. Both male and female monkeys showed dystrophin deficiency and muscle degeneration, like early Duchenne muscular dystrophy (DMD). These models allow DMD study without inheritance factors [75]. Furthermore, CRISPR-Cas9, coupled with SCNT, facilitated the generation of Guangxi Bama minipigs harboring heterozygous mutations in the SNCA gene, which encodes α-synuclein, implicated in Parkinson’s disease. The expression of mutated protein renders these pigs a valuable model for neurodegenerative disorder research [76].

### 3.2. CRISPR-Cas: Livestock Improvement

CRISPR-Cas9 modifications can enhance the breeding efficiency and overall well-being in farm animals, particularly their health. This technology has been successfully applied in various transgenic livestock species, including pigs, cattle, and fish [45,77].

Recent studies showed the in vitro potential of CRISPR-Cas9 in controlling viral diseases, which pose a global threat to meat production. While advances in vaccinology offer potential solutions, developing fully effective vaccines remains a substantial challenge. The targeted disruption or inactivation of key viral genes mediating infection presents a promising alternative. In pigs, the CRISPR-Cas9-mediated silencing of the *CD163* gene, encoding the Cluster of Differentiation 163 protein, was shown to significantly reduce porcine reproductive and respiratory syndrome virus (PRRSV) transmission, increasing pigs’ survival rates [78,79]. Similarly, the reduced incidence of classical swine fever virus (CSFV) and pseudorabies virus (PRV) infections in pigs was achieved through the CRISPR-Cas9-mediated introduction of a short hairpin RNA (shRNA) with antiviral activity [80]. Furthermore, the CRISPR-Cas9-mediated targeting of multiple loci within the African swine fever virus (ASFV) genome was employed to engineer a pig strain exhibiting acquired resistance to the virus [81]. Another application of the CRISPR-Cas9 method is the modulation of the animal immune system. The knockout of the PRNP and NRAMP1 genes, encoding cellular prion protein and natural resistance-associated macrophage protein 1, respectively, resulted in reduced susceptibility to tuberculosis and spongiform encephalopathy in cattle [82,83,84]. The inactivation of the TLR22 gene, which encodes toll-like receptor 22, suppressed the innate immune response in carp and enhanced disease resistance [85,86,87].

The CRISPR-Cas9 method enables the introduction of beneficial traits, directly contributing to livestock’s well-being by reducing stress, improving their adaptability to environmental changes, and minimizing the need for antibiotic use. The knock-in of the UCP1 gene, encoding uncoupling protein 1, enhanced non-shivering thermogenesis in brown adipose tissue, thereby improving cold tolerance in pigs. Furthermore, these transgenic pigs demonstrated enhanced lean meat production. Consequently, this genome editing offers a promising avenue for livestock production focused on improved meat quality [77].

Beyond improving animal well-being, CRISPR-Cas9 offers the potential to refine the dairy composition, leading to products with enhanced nutritional profiles, such as increasing the concentrations of beneficial proteins like casein or reducing the lactose content, or modified protein structures, ultimately contributing to both animal health and product quality [45,77,88]. In porcine meat production, CRISPR-Cas9 was utilized to increase the quantity and quality of meat by reducing the body fat percentage. It was achieved by knocking out the *MSTN* gene encoding for myostatin, leading to skeletal muscle hypertrophy in Erhualian pigs [89]. Moreover, CRISPR-Cas9 can potentially enhance muscle growth and prevent hindlimb weakness in pigs. Pigs engineered with a 40-base-pair deletion in exon 1 of the MSTN gene exhibited normal mobility, increased lean meat yields, and improved meat quality [90]. Another critical finding is that transgenic pigs expressing fatty acid desaturase-1, peroxisome proliferator-activated receptor γ, and PPARγ coactivator 1α, introduced by CRISPR-Cas9, produced meat enriched with polyunsaturated fatty acids, which offer significant cardiovascular and prenatal neural development benefits [66,91]. Additionally, CRISPR-Cas9-mediated modifications can yield hypoallergenically modified milk. Knockout of the α-lactalbumin (*LALBA*) and β-lactoglobulin (*BLG*) genes in goats resulted in milk with reduced immunogenic protein content, rendering it suitable for individuals with allergies [88].

Like those of mammals, the fish genome can be modified as well. CRISPR-Cas9 was used to alter the *MSTN* gene in catfish and flounder, which increased their growth and muscle mass. Changes in the *MSTN* provided catfish with an increase in body mass, muscle fibers, and fiber size [92]. Another significant direction in aquaculture breeding is the modification of fish phenotypes, particularly pigmentation; enhanced visual appeal correlates with increased market value. The precise targeting of genes regulating melanin synthesis, such as *slc45a2*, which is crucial in determining skin pigmentation, reduces tyrosinase activity and melanosome dysfunction, resulting in a uniform and stable phenotype and eliminating undesirable spotted pigmentation patterns. The economic benefits of these modifications manifest as a higher market price for fish with uniform and attractive coloration [93].

CRISPR-Cas9 technology offers significant potential to enhance farm animal breeding and well-being. It enables clear-cut genetic modifications to improve disease resistance, enhance physiological traits, and refine product quality. Similar applications in fish breeding focus on increasing growth and improving marketable traits like pigmentation.

### 3.3. CRISPR-Cas9: Production of Recombinant Proteins

CRISPR-Cas9 has transformed the production of recombinant proteins. While traditional systems relied on bacterial and yeast cells, these microbial platforms exhibit limitations in human protein production—notably the inability to perform critical post-translational modifications and the potential for immunogenic responses due to microbial antigens. Mammalian cell cultures offer the requisite post-translational modifications and present a competitive alternative to prokaryotic systems; however, they necessitate complex maintenance and incur substantial costs [94,95,96].

This problem can be solved through transgenic animals developed as bioreactors for recombinant protein production. Animals engineered using CRISPR-Cas9 facilitate efficient protein secretion in mammalian milk and avian egg whites. This approach simplifies protein purification while offering a scalable and cost-effective platform that ensures proper post-translational modifications [96,97].

Transgenic animals engineered for recombinant protein production are generated through various genetic modifications, including insertions and deletions. A prime example is the therapeutic knock-in (KI) of the human lactoferrin (LF) gene into the CSN1S1 locus, which encodes α-casein. This modification resulted in enhanced lactoferrin synthesis within the transgenic animal’s milk. With its inherent antimicrobial properties, the expressed lactoferrin provided crucial intestinal protection, significantly reducing piglet mortality [98]. CRISPR-Cas9 has also introduced human granulocyte–macrophage colony-stimulating factor (GM-CSF). The gene encoding GM-CSF was inserted into the CSN1S1 locus, which allows recombinant protein production in the mammary glands, simplifying the extraction and purification of GM-CSF [99]. Milk from transgenic sheep permits the production of melatonin. The arylalkylamine N-acetyltransferase (AANAT) and serotonin N-acetyltransferase (ASMT) genes, which control melatonin synthesis, are expressed only in the mammary gland due to the β-casein promoter [100].

Transgenic chickens are a notable example of recombinant protein production within egg whites. Specifically, the human interferon β (IFNB1) gene has been precisely integrated into the ovalbumin (OVA) locus, demonstrating the feasibility of generating protein-enriched egg whites [101,102]. This approach highlights the potential of transgenic chickens as bioreactors. Furthermore, the CRISPR-Cas9-mediated gene insertion of human monoclonal anti-HER2 antibodies into the OVA locus exemplifies another successful application, yielding therapeutic proteins exhibiting functional equivalence to the commercially available monoclonal antibody trastuzumab [102].

CRISPR-Cas9 overcomes the limitations of microbial systems and cell cultures by ensuring proper protein modification and simplifying purification, as demonstrated by the production of therapeutic proteins.

### 3.4. CRISPR-Cas9 and Xenotransplantation

The primary obstacle in organ transplantation is the severe shortage of donor organs, with the demand far exceeding the supply. This disparity has catalyzed the exploration of cross-species transplantation, or xenotransplantation [103]. The concept of interspecies organ transfer to humans traces back to 1667, marked by the initial attempt to transfuse sheep blood into human subjects. Subsequent experiments included a rabbit kidney transplant into a human in 1905 [104]. With the advent of advanced genetic modification technologies, like CRISPR-Cas9, transgenic animal models have emerged as promising candidates for organ donation.

Porcine models represent the predominant choice for xenotransplantation research. This is attributed to their advantageous biological characteristics, including a truncated gestation period, accelerated sexual maturation, and facile breeding. Furthermore, these animals exhibit a comparatively reduced risk of zoonotic disease transmission (infections transmitted from animals to humans) and demonstrate substantial physiological and anatomical congruence with human organ systems [61,66,105,106]. Investigations into the clinical applicability of porcine organs, encompassing the heart, kidney, integumentary tissue, cardiovascular structures, pancreas, liver, and lungs, are underway within xenotransplantation [106,107].

The utility of porcine models in xenotransplantation is constrained by immune rejection, mediated by specific porcine xenoantigens. However, CRISPR-Cas9 technology offers the possible ablation of these immunogenic determinants [108]. Genetic modifications facilitate the elimination of swine antigens, such as galactose-α1,3-galactose (α-Gal), cytidine-monophospho-N-acetylneuraminic acid, and beta-1,4-N-acetylgalactosaminyltransferase 2. The targeted deletion of these xenoantigens through CRISPR-Cas9 allowed the attenuation of human IgM and IgG binding to porcine peripheral blood mononuclear cells (PBMCs), thereby mitigating the immune response and enhancing the clinical xenotransplant compatibility [61,109,110].

In addition to immune compatibility, minimizing zoonotic risks remains a challenge in xenotransplantation [66]. Porcine zoonotic pathogens, representing a significant concern in xenotransplantation, encompass porcine endogenous retroviruses (PERV), porcine cytomegalovirus (PCMV), porcine lymphotropic herpes virus (PLHV), and hepatitis E virus (HEV). Utilizing CRISPR-Cas9 to remove these viral elements from the porcine genome substantially reduces the potential for cross-species viral transmission [61]. In 2015, CRISPR-Cas9 was successfully employed to inactivate PERV, resulting in a reduction in transmission from porcine to human cells of over 1000 times [111].

Despite these significant advancements, ongoing improvements in the current genetic engineering methods remain imperative to expand the donor pool and ensure equitable access to suitable organs for all patients requiring transplantation. CRISPR-Cas9 enables genetically modified animals to be potential transplant donors. While immune rejection and zoonotic risks are challenges, CRISPR-Cas9 is used to eliminate animals’ antigens and inactivate viruses. Continued progress in genetic engineering is needed to increase organ availability.

## 4. Discussion

The CRISPR-Cas9 system has reformed genetic engineering, becoming a leading tool for targeted genome editing. Its capacity to introduce precise modifications within the DNA of transgenic animals has garnered significant attention, particularly in livestock breeding [4]. While the potential benefits of CRISPR-Cas9 in enhancing livestock traits such as disease resistance, meat quality, and growth rates are substantial, the transition from laboratory research to large-scale, commercial implementation is fraught with both opportunities and multifaceted challenges.

CRISPR-Cas9 heralds a revolution in livestock farming, unlocking a cumulative beneficial impact and directly translating to enhanced animal health, productivity, and overall well-being. We have witnessed transformative impacts across key areas by precisely engineering desired traits into farm animals’ genomes. Through targeted genetic modifications, CRISPR-Cas9 facilitates heightened productivity and superior food quality by accelerating growth, enhancing digestive efficiency, increasing egg production, enabling leaner meat production, and optimizing the composition of dietary products to meet specific nutritional needs. Simultaneously, this technology fortifies animal health and welfare, providing robust resistance to diseases, including zoonotic infections, and improving the tolerance to environmental stressors like low temperatures. Furthermore, CRISPR-Cas9 accelerates biopharmaceutical production, enabling the efficient synthesis of therapeutics within the mammary glands or avian egg whites, thereby increasing the production efficiency and lowering costs [77,82,83,84,88,96].

CRISPR-Cas9 has advanced biomedical research by enabling the rapid generation of diverse and tailored animal models for a broad spectrum of human diseases. These models are powerful tools in investigating disease pathogenesis, testing therapeutic interventions, and advancing our understanding of human biology [70,75,76]. Biomedical research frequently involves single-sex animal models, particularly in preclinical studies focused on sex-specific biological processes such as oogenesis, spermatogenesis, sex-specific cancers, Y chromosome biology, and X chromosome inactivation. This practice raises ethical concerns due to the required elimination of the opposite sex. Similarly, industrial sectors, such as dairy and meat production, rely on breeding animals of specific sexes, leading to both ethical and economic implications. Consequently, the development of reliable methods for the generation of single-sex litters is crucial. CRISPR-Cas9 technology offers a potential solution. A recent study showed that CRISPR-Cas9 can produce single-sex mouse litters. By implementing a two-component system targeting an essential gene, researchers achieved exclusive male or female offspring production, indicating potential for use in livestock and avian species [112].

CRISPR-Cas9 may also play a pivotal role in disease prevention by reducing the transmission of infectious agents from animals to humans. The genetic modification of disease-carrying organisms, such as the yellow fever mosquito (*Aedes aegypti*), can potentially limit the spread of diseases like malaria, dengue fever, chikungunya, and Zika [113,114].

While CRISPR-Cas9 offers numerous advantages, its application is not without inherent risks, and evidence from animal models suggests that these risks can have significant consequences. Notably, off-target mutations, or unintended genetic alterations stemming from mismatches between the sgRNA and non-target DNA sequences, have been observed to have significant genomic-level consequences [113,114]. Often, these mutations stem from mismatches between the single-guide RNA (sgRNA) and non-target DNA sequences; off-target mutations can manifest as large deletions, genomic rearrangements, and functional gene disruptions [115,116,117]. For example, studies in mice have demonstrated that, even with optimized sgRNA design, off-target cleavage can lead to large deletions at unexpected genomic loci, resulting in observable phenotypic abnormalities [118,119]. Furthermore, genomic rearrangements, including large structural variants at both on- and off-target sites, have been reported in zebrafish following CRISPR-Cas9 editing, highlighting the potential for complex and unintended genomic instability [117].

The frequency of these mutations is particularly problematic in mammals, characterized by their complex genomes and abundant protospacer adjacent motif sites [120]. While off-target effects may be mitigated in species with short reproductive cycles, they pose a substantial challenge in livestock exhibiting prolonged maturation periods, where the long-term consequences of subtle off-target changes might only become apparent in subsequent generations. Mitigating these risks demands advancements in precision editing techniques and the implementation of the rigorous pre-screening of genetic targets using bioinformatics tools [121].

Beyond off-target mutations, CRISPR-Cas9 applications present significant challenges linked with the potential for mosaicism, wherein multiple distinct genotypes accumulate within a single individual. This phenomenon has been directly observed in livestock models, arising from variable gene modifications at different developmental stages during embryogenesis. Namely, studies in cattle have shown significant variation in the efficiency of gene editing across different tissue types within the same animal, leading to a mosaic pattern of gene modification that complicates phenotypic analysis and potentially impacts the efficacy and safety of the intended genetic alteration [122,123]. This coexistence of genetically heterogeneous cell populations within the organism poses challenges in ensuring consistent and predictable outcomes in gene-edited animals [121,124].

Introducing transgenic organisms, such as genetically modified mosquitoes, into environmental ecosystems carries the risk of altering predator–prey dynamics and disrupting natural food chain structures, potentially leading to unforeseen ecological consequences [113,114]. Animal model studies in controlled environments have simulated these effects, demonstrating, for example, the rapid decline of native insect populations following the introduction of gene-drive modified mosquitoes with a fitness advantage [125]. These findings underscore the potential for significant and potentially irreversible ecological impacts.

Furthermore, the widespread adoption of CRISPR-Cas9 is constrained by the substantial costs of generating transgenic livestock and the stringent regulatory frameworks governing genetically modified organisms (GMOs) [126]. The regulatory frameworks governing the application of gene editing in genetically modified animals exhibit considerable heterogeneity across international jurisdictions. In the United States, the Food and Drug Administration (FDA) conducts the primary regulatory oversight of genetically modified animals, including those modified using CRISPR-Cas9. The FDA classifies intentional genomic alterations (IGAs) in animals as new animal drugs under the Federal Food, Drug, and Cosmetic Act, necessitating developers to demonstrate safety for the animal, the environment, and, where applicable, human consumption [127]. Conversely, the European Union maintains a more stringent regulatory framework for genome-edited organisms. A 2018 Court of Justice of the European Union (CJEU) ruling determined that organisms obtained through mutagenesis techniques, including CRISPR-Cas9, fall under the purview of Directive 2001/18/EC, which regulates the deliberate release of GMOs into the environment. This decision mandates comprehensive risk assessments, labeling, and monitoring protocols for CRISPR-edited animals, aligning them with traditionally defined GMOs [128]. China has actively pursued the development and application of CRISPR-Cas9 technology in both agriculture and biomedicine. The Ministry of Agriculture and Rural Affairs (MARA) has issued guidelines that differentiate genome-edited organisms from traditional GMOs, particularly in cases where no foreign DNA is introduced. While specific regulations for genome-edited animals are still in development, China’s regulatory approach emphasizes innovation, biosafety, and alignment with national strategic priorities. The divergent regulatory landscapes for CRISPR-Cas9 applications in genetically modified animals reflect varying policy priorities and risk assessment paradigms. The United States adopts a product-based approach centered on safety and efficacy, the European Union enforces a process-based framework with rigorous requirements, and China prioritizes innovation within a structured regulatory context [129]. Understanding these differences is crucial for stakeholders navigating the global landscape of animal biotechnology.

The commercialization of genetically modified livestock raises profound ethical and ecological concerns. The large-scale industrial farming of genetically engineered animals and the subsequent commercialization of transgenic animal models and their products may destabilize and undermine conventional agricultural practices and compromise overall biodiversity [130,131,132]. Moreover, ethical considerations extend to modifying embryos and zygotes, as these interventions may introduce latent and undetected genetic alterations with long-term, population-level impacts.

Thus, given the diverse and far-reaching applications of CRISPR-Cas9, rigorous regulatory oversight is imperative to mitigate these multifaceted risks, ensuring the responsible and sustainable implementation of this powerful technology.

## 5. Conclusions

CRISPR-Cas9 stands out as a transformative genome editing technology, offering significant advantages in terms of simplicity, efficiency, and affordability compared to earlier platforms such as zinc finger nucleases (ZFNs) and transcription activator-like effector nucleases (TALENs). The broad spectrum of applications of CRISPR-Cas9 has driven significant advancements in agricultural and biomedical research. In the farming sector, this technology has been used to generate transgenic livestock, addressing challenges in food production and improving animal health and welfare. Within biomedical sciences, CRISPR-Cas9 has developed more accurate preclinical models of human diseases and facilitated the production of recombinant proteins and potential xenotransplantation therapies. Despite these successes, however, significant research gaps persist. Concerns about off-target effects, mosaicism, and the delivery efficiency underscore the continued need for improved genome editing precision. To address these limitations, future research should prioritize further developing and optimizing emerging tools such as base editors and prime editing. These offer promising avenues for more targeted and predictable genetic modifications. Furthermore, a critical area for future investigation lies in advancing in vivo animal models that can more accurately recapitulate human physiology and disease, thereby accelerating the translation of CRISPR-Cas9-based therapies into clinical practice. Establishing clear and harmonized regulatory frameworks will also be crucial to ensure the safe and ethical application of gene editing technologies in agricultural and medical contexts. Ultimately, continued innovation in these areas will be essential to fully unlock the transformative potential of CRISPR-Cas9 while mitigating its inherent risks.

## Figures and Tables

**Table 1:** Comparison of CRISPR-Cas9 acting in bacteria and eukaryotic cells.

Aspect	CRISPR-Cas9 in Bacteria	CRISPR-Cas9 in Eukaryotic Cells
Function	Adaptive immune system, protecting bacteria against viral infection	Genome editing tool, allowing DNA modification in living organisms
RNA components	CRISPR RNA (crRNA) and trans-activating CRISPR RNA (tracrRNA) exist as two separate molecules	crRNA and tracrRNA are combined into a synthetic sgRNA
Protein localization	Cas9 functions in bacterial cytoplasm	Bioengineered Cas9 with nuclear localization signal (NLS), which allows transport into cell nucleus
Codon utilization	Matches bacterial codon	Codon is optimized for expression in eukaryotic cells
Target specificity	Targets foreign DNA based on spacer sequences	Synthetic sgRNA designed to direct Cas9 to specific DNA sequence site
Delivery mechanism	Naturally occurring mechanisms	Delivery through microinjection, electroporation, or viral vectors
Repair mechanisms	While CRISPR-Cas9 in bacteria primarily cleaves foreign DNA, creating DSBs, these breaks are subsequently repaired by the bacteria’s DNA repair mechanisms; thus, CRISPR-Cas9 indirectly triggers these repair pathways	NHEJ or HDR-driven genome modifications

**Table 2:** Methods of CRISPR-Cas9 delivery.

Type	Mechanism	Mode of Action	Examples of Genetically Modified Animals
Physical	Electroporation	Short electrical pulses create transient pores in the cell membrane, enabling CRISPR-Cas9 entry [30,31].	Mammals: Pig [20,32,33,34], Cattle [20,35], Mouse [20,21,36], Rat [19,37], Goat [38] Non-mammals: Zebrafish [31]
	Microinjection	CRISPR-Cas9 is microinjected into fertilized egg pronuclei, and embryos are transferred to pseudopregnant surrogates for development [39].	Mammals: Mouse [40,41], Rabbit [42], Cattle [43], Pig [18,32,44], Sheep [17,45] Non-mammals: Lizard [46], Zebrafish [47,48], Catfish [49]
Chemical	Nanoparticles	Nanocarrier-mediated delivery, utilizing lipid, gold, polymer-coated nanoparticles, and exosomes, facilitates direct CRISPR-Cas9 cargo transfer to target cells [23].	Mammals: Rat [50], Mouse [24,51,52,53,54]
Viral	Adenoviruses (AVs) Adeno-associated viruses (AAVs) Lentiviruses (LVs)	Viral vectors deliver CRISPR-Cas9 to host cells. Viruses are modified for safe gene delivery by disabling replication [55].	Mammals: Pig [56], Canine [57], Mouse [25,26], Rat [22,58]
Somatic cell nuclear transfer (SCNT)	SCNT + microinjection	Involves screening of somatic cells for gene alterations caused by CRISPR-Cas9 microinjection. After identification, altered nuclei are transferred into enucleated oocytes [27].	Mammals: Mouse [59], Pig [27,29]

## Data Availability

No new data were created or analyzed in this study. Data sharing is not applicable to this article.

## Abbreviations

The following abbreviations are used in this manuscript:

α-Gal	Galactose-α-1,3-galactose
AANAT	Arylalkylamine N-acetyltransferase
AAV	Adeno-associated virus
ASFV	African swine fever virus
ASMT	Serotonin N-acetyltransferase
AV	Adenovirus
BLG	β-Lactoglobulin
Cas9	CRISPR-associated protein 9
CD163	Cluster of Differentiation 163 protein
CDK2	Cyclin-dependent kinase 2
CJEU	Court of Justice of the European Union
CMAH	Cytidine-monophospho-N-acetylneuramine
CRISPR	Clustered Regularly Interspaced Short Palindromic Repeats
crRNA	CRISPR RNA
CSFV	Classical swine fever virus
CSN1S1	Casein-alpha 1
DMD	Duchenne muscular dystrophy
DNA	Deoxyribonucleic acid
DSB	Double-strand break
E-HEV	Hepatitis E virus
E2F1	E2F Transcription Factor 1
E7	E7 protein
FAH	Fumarylacetoacetate hydrolase
FDA	Food and Drug Administration
GM-CSF	Granulocyte–macrophage colony-stimulating factor
GMO	Genetically modified organism
HCC	Hepatocellular carcinoma
HDR	Homology-directed repair
HNH	Histidine–asparagine–histidine endonuclease domain
HPV	Human papilloma virus
HTI	Hereditary tyrosinemia type I
HTT	Huntingtin
IFNB1	Interferon-β
IGA	Intentional genomic alteration
KI	Knock-in
KO	Knock-out
LALBA	α-Lactalbumin
LF	Lactoferrin
LV	Lentivirus
MARA	Ministry of Agriculture and Rural Affairs
MSTN	Myostatin
NHEJ	Nonhomologous end joining
NLS	Nuclear localization signal
NRAMP1	Natural resistance-associated macrophage protein 1
OVA	Ovalbumin
PAM	Protospacer adjacent motif
PBMC	Peripheral blood mononuclear cells
PCMV	Porcine cytomegalovirus
PERV	Porcine endogenous retrovirus
PGC1α	PPARγ coactivator 1α
PLHV	Porcine lymphotropic herpes virus
PPARγ	Peroxisome proliferator-activated receptor γ
PRNP	Cellular prion protein
PRRSV	Porcine reproductive and respiratory syndrome virus
PRV	Pseudorabies virus
PUFA	Polyunsaturated fatty acid
RB	Retinoblastoma protein
RGEN	RNA-guided endonuclease
RNA	Ribonucleic acid
RNAi	RNA interference
RuvC	Crossover junction endodeoxyribonuclease RuvC
SCNA	α-Synuclein
SCNT	Somatic cell nuclear transfer
SFAT-1	Synthesized fatty acid desaturase-1
sgRNA	Single-guide RNA
shRNA	Hairpin structure RNA
TALENs	Transcription activator-like effector nucleases
TLR22	Toll-like receptor 22
tracrRNA	Trans-activating CRISPR RNA
UPC1	Uncoupling protein 1
ZFNs	Zincfinger nucleases
α-Gal	Galactose-∝1,3-galactose
